# Preparation of Nickel Nanoparticles by Direct Current Arc Discharge Method and Their Catalytic Application in Hybrid Na-Air Battery

**DOI:** 10.3390/nano8090684

**Published:** 2018-09-01

**Authors:** Fengmei Su, Xuechao Qiu, Feng Liang, Manabu Tanaka, Tao Qu, Yaochun Yao, Wenhui Ma, Bin Yang, Yongnian Dai, Katsuro Hayashi, Takayuki Watanabe

**Affiliations:** 1 Faculty of Metallurgical and Energy Engineering, Kunming University of Science and Technology, Kunming 650093, China; 15365602721@163.com (F.S.); qiuxuechao183@163.com (X.Q.); qutao_82@126.com (T.Q.); yaochun9796@163.com (Y.Y.); mwhsilicon@126.com (W.M.); kgyb2005@126.com (B.Y.); daiyn@cae.cn (Y.D.); 2 State Key Laboratory of Complex Nonferrous Metal Resources Clear Utilization, Kunming University of Science and Technology, Kunming 650093, China; 3 Department of Chemical Engineering, Kyushu University, Fukuoka 819-0395, Japan; mtanaka@chem-eng.kyushu-u.ac.jp (M.T.); watanabe@chem-eng.kyushu-u.ac.jp (T.W.); 4 Department of Applied Chemistry, Faculty of Engineering, Kyushu University, Fukuoka 819-0395, Japan; k.hayashi@cstf.kyushu-u.ac.jp

**Keywords:** nickel nanoparticle, arc discharge method, arc-anode attachment, catalyst, Na-air battery

## Abstract

Nickel nanoparticles were prepared by the arc discharge method. Argon and argon/hydrogen mixtures were used as plasma gas; the evaporation of anode material chiefly resulted in the formation of different arc-anode attachments at different hydrogen concentrations. The concentration of hydrogen was fixed at 0, 30, and 50 vol% in argon arc, corresponding to diffuse, multiple, and constricted arc-anode attachments, respectively, which were observed by using a high-speed camera. The images of the cathode and anode jets were observed with a suitable band-pass filter. The relationship between the area change of the cathode/anode jet and the synchronous voltage/current waveform was studied. By investigating diverse arc-anode attachments, the effect of hydrogen concentration on the features of nickel nanoparticles were investigated, finding that 50 vol% H_2_ concentration has high productivity, fine crystallinity, and appropriate size distribution. The synthesized nickel nanoparticles were then used as catalysts in a hybrid sodium–air battery. Compared with commercial a silver nanoparticle catalyst and carbon black, nickel nanoparticles have better electrocatalytic performance. The promising electrocatalytic activity of nickel nanoparticles can be ascribed to their good crystallinity, effective activation sites, and Ni/NiO composite structures. Nickel nanoparticles prepared by the direct current (DC) arc discharge method have the potential to be applied as catalysts on a large scale.

## 1. Introduction

Thermal plasma enables rapid evaporation rate, abrupt temperature gradients, and high chemical reaction, and is therefore a fascinating route for the fabrication of nanomaterials. Using direct current (DC) arc plasma for nanomaterial production has several advantages. First, this method is an environmentally friendly technique because DC arc plasmas do not generate toxic by-products or hazardous gases. Second, DC arc plasma can be used for melting refractory metals because of its high temperature. Finally, a high purity nanoparticle can be continuously produced. The DC arc plasma has been used for the synthesis of various nanomaterials, such as carbon [1,2,3], alloy [4,5], oxide [6,7], intermetallic compound [8], and high-purity metal nanopowders [9,10]. 

In the arc discharge process, the raw material is offered by the anode when preparing the nanoparticles, and the productivity of the nanoparticle depends on the anode consumption rate. Accordingly, the anodic region and anode phenomena need to be further analyzed to increase the yield and control nanoparticle size. In fact, the anode boundary has three typical arc-anode adhesion modes, namely, diffusion, multiple, and constricted attachments [11,12]. Different arc-anode attachment modes contribute to the competition between the cathode and anode jets. Nevertheless, an in-depth research of the arc-anode attachment modes in argon-hydrogen arcs for nanoparticle production has not been conducted, and thus the arc discharge method is rarely used in nanoparticle production.

Considerable attention has been drawn to nickel nanoparticles due to their excellent chemical, physical, and electronic properties [13,14]. Recent research tendencies have focused on energy conversion and storage devices including metal-air batteries, fuel cells and supercapacitors, in which the supercapacitors are characterized by their high performance in terms of energy and power density, simple operating conditions and long cycling life [15,16,17]. Besides, Na-air batteries have gained much attention as next generation power storage because of their high theoretical specific density and capacity. Nickel nanoparticles are potential catalysts due to their large surface areas and additional active sites. Nickel nanoparticles catalyze the transfer hydrogenation of olefins and carbonyl compounds and the reductive amination of aldehydes with 2-propanol as hydrogen donor [18]. However, the use of nickel nanoparticles as catalysts for the oxygen reduction reaction (ORR) and oxygen evolution reaction (OER) for metal-air batteries is rarely reported. Recently, hybrid sodium-air batteries have potential application in electric vehicles due to their high energy density and low cost [19,20]. Therefore, developing a cheap non-noble metal catalyst for hybrid sodium-air battery is necessary. 

In the present work, nickel nanoparticles were prepared through DC arc discharge, and diffusion, multiple, and constricted attachment modes were observed in argon-hydrogen arcs. The effect of arc-anode attachment mode on the diameter of nanoparticles and their size distribution is investigated by using a high-speed camera and current/voltage measurement. Nickel nanoparticle generated by DC arc discharge in 50% H_2_ concentration has high productivity, fine crystallinity, and appropriate size distribution. Therefore, the nickel nanoparticles prepared under 50 vol% H_2_ were used in hybrid sodium-air batteries and displayed good catalytic performance, which is comparable to commercial silver nanoparticles.

## 2. Experimental

**Figure 1:**
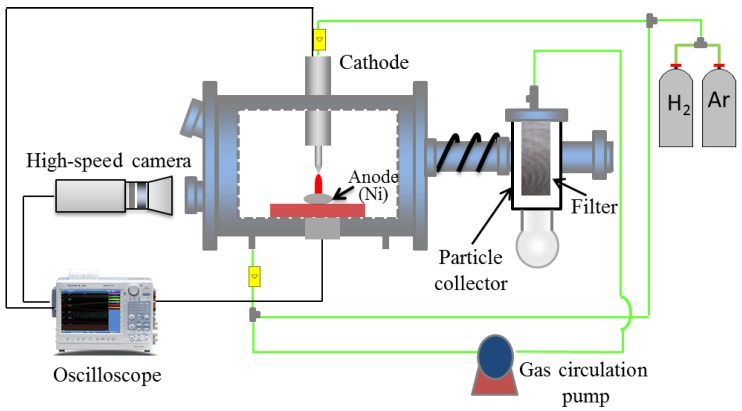
Schematic of the experimental setup for nickel nanoparticle production by direct current (DC) arc discharge method.

Figure 1 shows a schematic diagram of the experimental device used for preparing nickel nanoparticles and a synchronized high-speed camera for arc behavior observation. The setup is composed of a power source, an arc discharge chamber, a particle collector, and a gas circulator. Argon and/or hydrogen plasma gases were introduced to the system after the arc discharge chamber was evacuated. The anode was a cylindrical nickel ingot, which was placed on the water-cooled copper. After the arc ignition, metal fume was formed from the surface of the raw material. The nanoparticles grow after nucleation, coagulation, and collision [5]. There was no gas circulation during the experiment. Synthesized nanoparticles were collected at the filter by using a gas circulation pump.

### 2.1. Nickel Nanoparticle Preparation 

The experimental parameters are as follows. The ingot using 40 g of nickel (99.9%, Nilaco, Ltd., Tokyo, Japan) as anode was placed on a water-cooled copper. A cathode rod added with tungsten at 2 wt% thoria with a diameter of 6 mm was placed diagonally from the anode. The arc was formed for 12 min in Ar and the mixture of Ar-H_2_ gas at the pressure of 101.325 kPa. We controlled the arc current at 100 A and electrode gap distance at 6 mm. The concentration of hydrogen was fixed at 0, 30, and 50 vol% in argon arc. The anode and cathode jets are observed successfully by using band-pass filters of 656 ± 5 nm and 500 ± 5 nm, which are installed on the high-speed camera (FASTCAM-SA WTI, Photron, Tokyo, Japan), respectively. The optical system for cathode and anode jet observation and the synchronization observation between cathode/anode jet and current/voltage refer to our previous work [3,10].

### 2.2. Catalyst Preparation for Hybrid Na-Air Battery 

A hybrid sodium-air battery consists of a sodium metal anode, 1 M NaClO_4_ in ethylene carbonate/dimethyl carbonate (1:1) with 1 vol% of fluoroethylene carbonate as anolyte, a solid conductor with Na_3_Zr_2_Si_2_PO_12_ (ionic conductivity of 1.3 × 10^−3^ S cm^−1^ at 25 °C) [19], 1 M NaOH as catholyte, and an air electrode containing nickel nanoparticles as catalysts. During the discharge process, the oxygen in air diffuses to the catalytic site. Then, oxygen is reduced on the cathode electrolyte/catalyst (nickel nanoparticles)/air three-phase interface and combines with H_2_O and electrons in the cathode to form OH^−^ [21].

For comparison, three different catalyst inks were prepared by sonicating 15 mg of nickel nanoparticles, silver nanoparticle (99.5%, Macklin, Shanghai, China), and carbon black (Ebory, Tianjin, China) in a mixture of ethanol and deionized water (2 mL; 1:1) with a drop of 5% polytetrafluoroethylene as binder. The resulting mixtures were then under continuous stirring for 30 min. The catalyst inks were then dripped onto the gas diffusion layer [22], and the catalyst layer on the carbon paper was treated under a pressure (8 MPa) to obtain the air electrodes [21]. The catalyst loading in the air electrodes was 1 mg/cm^2^. The assembled Na-air battery was tested at 30 °C in atmospheric air (Figure S1). A LAND battery tester (CT2001A, Wuhan LAND Electronics, Wuhan, China) was used for charge and discharge tests. 

### 2.3. Characterization

The morphologies of the samples were examined by transmission electron microscopy (TEM). The TEM study was performed under a JEM-2100 microscope (JEOL, Osaka, Japan). The electron accelerating voltage was 200 kV. The structure and chemical properties were studied with X-ray diffraction (XRD) and X-ray photoelectron spectroscopy (XPS). The phase identification of the prepared nickel nanoparticles was measured by the XRD (MXP3TA, Mac Science, Beijing, China) operation of the Cu Kα source (k = 0.1541 nm). XPS data were collected using a Kα^+^ X-ray photoelectron spectrometer. Spectra obtained from 1486.6 eV photon beams selected from mono Al Kα source were obtained.

## 3. Results and Discussion

**Figure 2:**
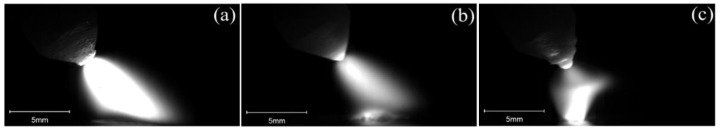
Snapshots of high-speed camera for argon arc with different hydrogen concentrations: (**a**) 0, (**b**) 30, and (**c**) 50 vol%.

Figure 2 shows the snapshots of a high-speed camera of argon arc with different H_2_ concentrations of 0, 30, and 50 vol%, corresponding to Figure 2a–c, respectively. We assumed that the cathode and anode jets are composed of hydrogen and nickel, respectively. Three typical arc-anode attachment modes were observed in argon arc at different H_2_ concentrations. Figure 2a illustrates that the cathode jet impingements on the anode surface form a stagnation layer in front of the anode, which contributes to the well-known bell shape of the arc. In this case, the anode arc root is rather diffuse. This arc-anode attachment mode is expressed as a diffusion attachment mode [12]. Several small anode spots at the anode boundary can be seen at the same time in Figure 2b, which is the feature of multiple attachment modes [13]. Figure 2c presents the constricted attachment mode. The original reason of different arc-anode attachment modes is the evaporation of anode. The competition of the cathode jet and the anode jet leads to different forms of arc-anode attachment mode [10]. Multiple attachment modes have been considered to be transition modes between diffuse and contractile modes. The transition of the attachment mode is mainly related to the variation of anode evaporation rate caused by different hydrogen concentrations. 

**Figure 3:**
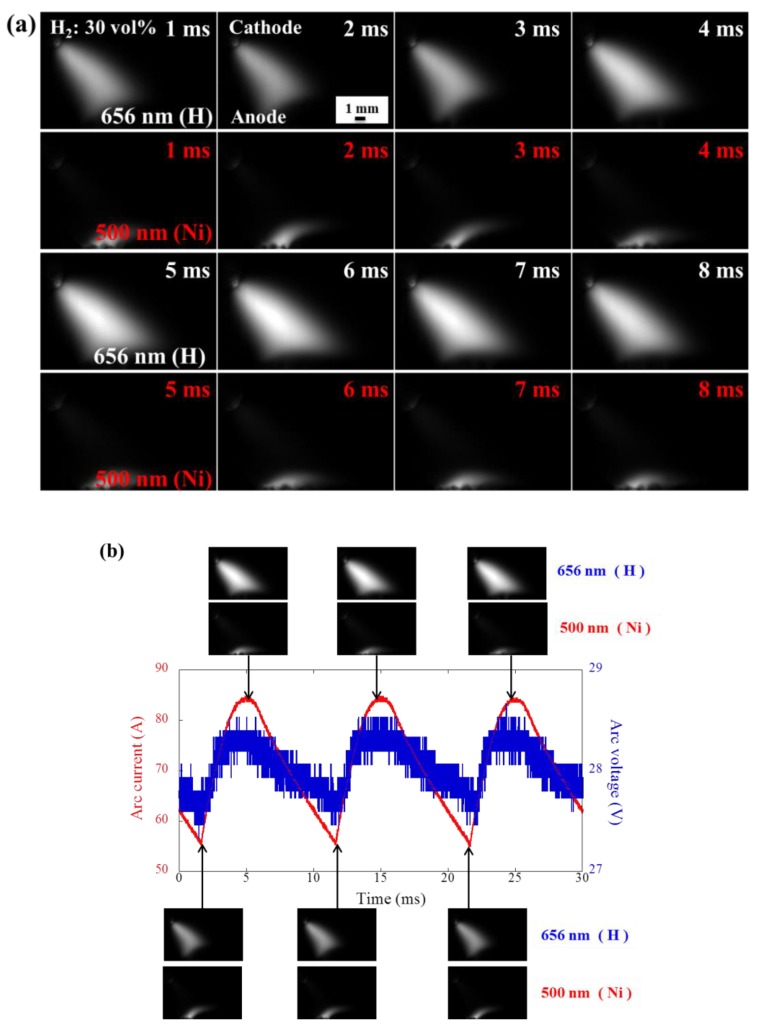
(**a**) Representative snapshots of a high-speed camera in argon arc when hydrogen concentration is 30 vol%. H emission is the upper one, and Ni emission is the lower one; (**b**) corresponding voltage and current waveform of argon arc synchronized with the snapshots of H and Ni emissions.

No anode jet was observed in pure Ar conditions, and thus we used a high-speed camera with the band-pass filter to observe the cathode and anode jets when the hydrogen concentrations were 30 and 50 vol%. Figure 3a shows the snapshots of the high-speed camera of argon arc when hydrogen concentration is 30 vol%. The cathode jet is on the top of anode jet in this figure. From these snapshots, the multiple attachments can be concluded to form at the anode jet region with 30 vol% of hydrogen. These images are used for the illustration of the variation in the anode and cathode jet areas. Figure 3b exhibits the current and voltage waveforms synchronized with snapshots. In multiple attachment modes, the area of the hydrogen cathode jet at the peak value of the arc current is larger than that of the arc valley. On the contrary, the hydrogen anode jet is larger at the valley of the arc current than that at the peak of the arc current. The reason is that the high arc current is helpful in enhancing the cathode jet, but the anode jet decreases due to the balance between the cathode and anode jets. 

**Figure 4:**
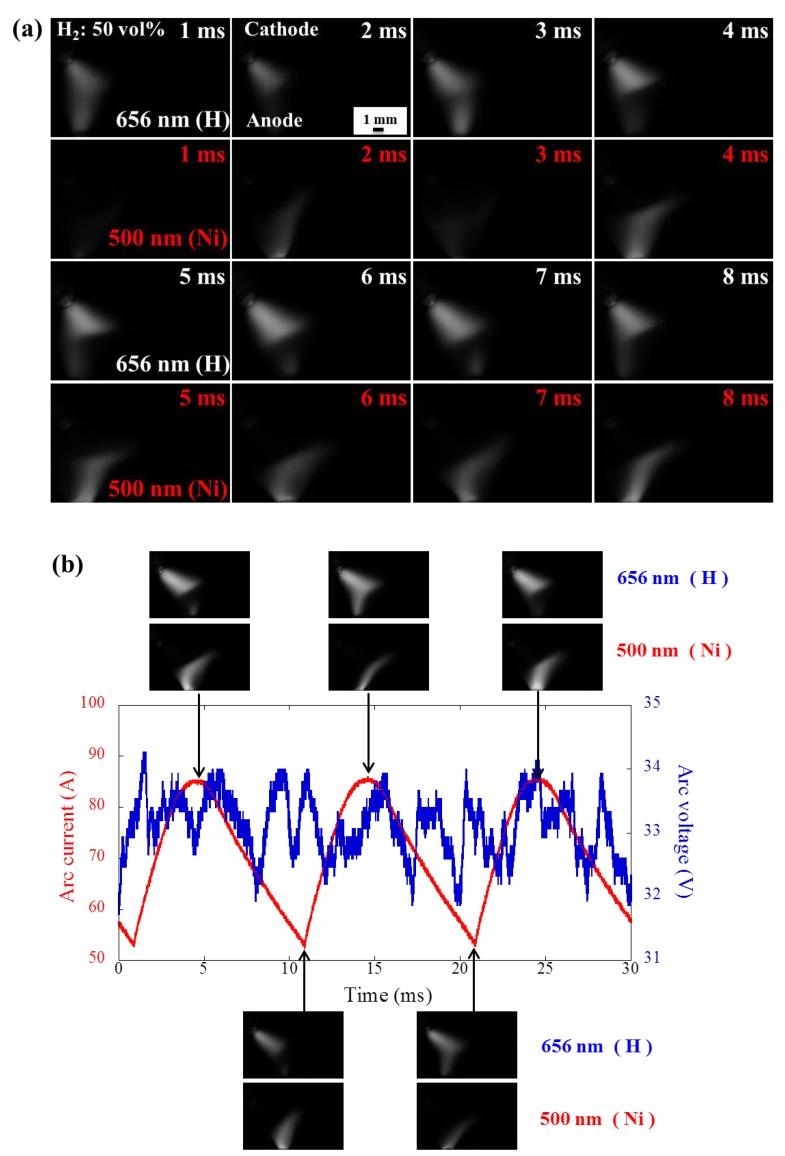
(**a**) Representative snapshots of the high-speed camera in argon arc when hydrogen concentration is 50 vol%. H and Ni emissions are the upper and lower ones, respectively; (**b**) Corresponding voltage and current waveform of argon arc synchronized with the snapshots of H and Ni emissions.

Figure 4a shows the snapshots of the high-speed camera for argon arc with 50 vol% of hydrogen. Constricted mode can be clearly seen at the anode boundary. The area of the nickel anode jet increases with increasing hydrogen concentration, whereas the hydrogen cathode jet area drops with increasing hydrogen concentration due to the strong intensity of the cathode jet. This result can be explained by the competition between cathode jet and anode jet [10]. Figure 4b presents the current and voltage waveforms of argon arc with 50 vol% of hydrogen synchronized with the arc behavior snapshots. Noticeably, the relatively large nickel anode jet is observed at the peak of the arc current. In addition, significant movement of anode spot can be seen in the constricted mode, which is also confirmed by the large fluctuation of voltage waveform. However, no clear relationship is observed between the arc current and the area of the anode jet due to the instability of the arc. 

**Figure 5:**
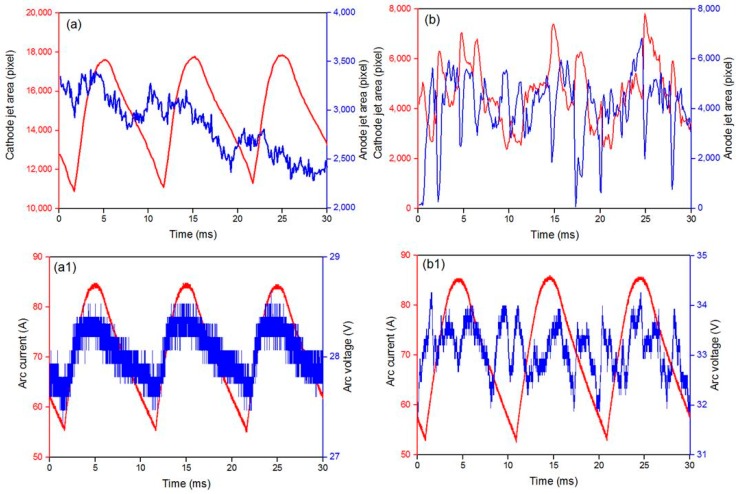
Waveform of anode/cathode jet area variation (upper) and synchronized arc current and voltage waveforms (lower) for argon arc with different hydrogen concentrations. (**a**,**a1**) and (**b**,**b1**) correspond to the hydrogen concentrations of 30 and 50 vol%, respectively.

Figure 5 shows the relationship between the anode and cathode jet areas (upper) of argon arc with different hydrogen concentrations and the time variation of the current and voltage waveforms of synchronous arc (lower). Figure 5a-a1 and b-b1 illustrate the hydrogen concentrations of 30 and 50 vol%, respectively. 

In the waveform, the peak of cathode jet area variation and the valley of the anode jet area variation appear nearly simultaneously. The average areas of the cathode jet are 2850 and 4000 pixels, corresponding to the hydrogen concentrations of 30 and 50 vol%, respectively. Figure 5 show that the waveform of the cathode jet variation follows the shape of the arc current in multiple attachment modes at 30 vol% of hydrogen. By contrast, no clear relationship between the arc current and the area of the anode jet in the constricted mode is found. The variations in the cathode and anode jet areas show large fluctuations in the constricted mode, and these fluctuations are attributed to the instability of the arc. In addition, the noise in voltage waveform can be explained by the instability of the arc. Hydrogen concentration plays a critical role in the formation of different attachment modes, and a high hydrogen concentration contributes to the formation of constricted mode due to the large evaporation rate.

**Figure 6:**
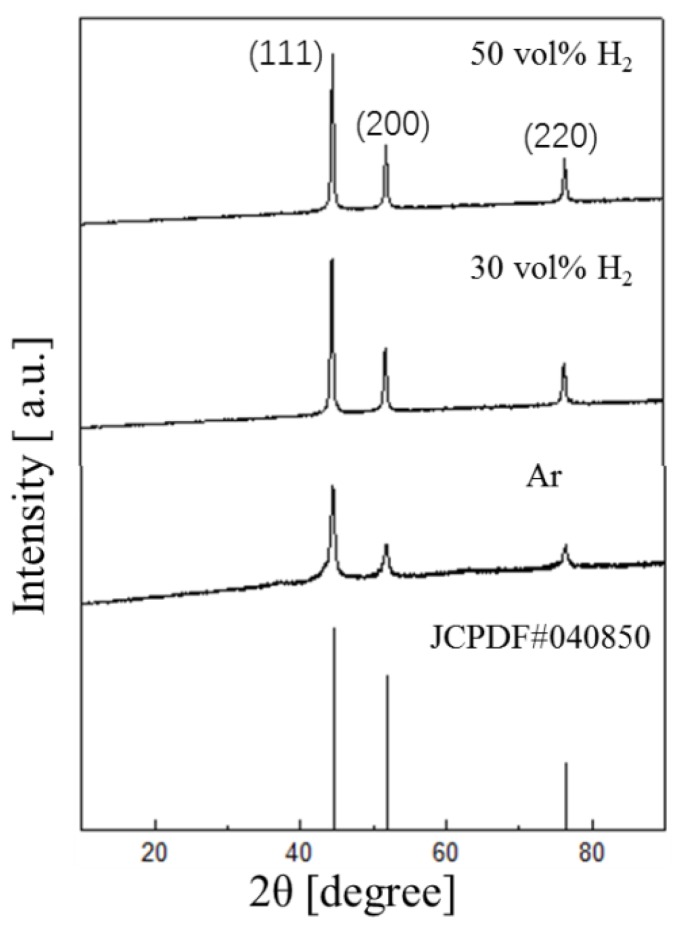
XRD patterns of nickel nanoparticle in argon arc at different hydrogen concentrations.

Figure 6 presents the XRD patterns of the synthesized nickel nanoparticles in the argon arc with different hydrogen concentrations. Three typical diffraction peaks of the patterns can be fully indexed into (111), (200), and (220) planes of nickel. The diffraction peaks of the nickel nanoparticles generated in 100 vol% Ar can be found to be weak and broad, which indicates a weak crystallinity of the nickel nanoparticles with a relatively small diameter. With the increasing concentration of hydrogen, the diffraction of peaks becomes sharper and stronger, indicating an improved crystallinity and large particle size. When the hydrogen concentration is 50 vol%, the diffraction of peaks is the sharpest and strongest, contributing to the best crystallinity of nickel nanoparticles with relatively large particle size. According to Scherrer equation, the average grain size of based on the (111) diffraction peaks for the samples with Ar, 30 vol% H_2_, 50 vol% H_2_ are 20 nm, 34 nm, 59 nm, respectively. Compared with other outputs of 0.01 g/min, 0.03 g/min at the hydrogen concentration of 0 vol% and 30 vol%, the highest output of 0.3 g/min is obtained when the hydrogen concentration is 50 vol%, because a high hydrogen concentration leads to high evaporation. 

**Figure 7:**
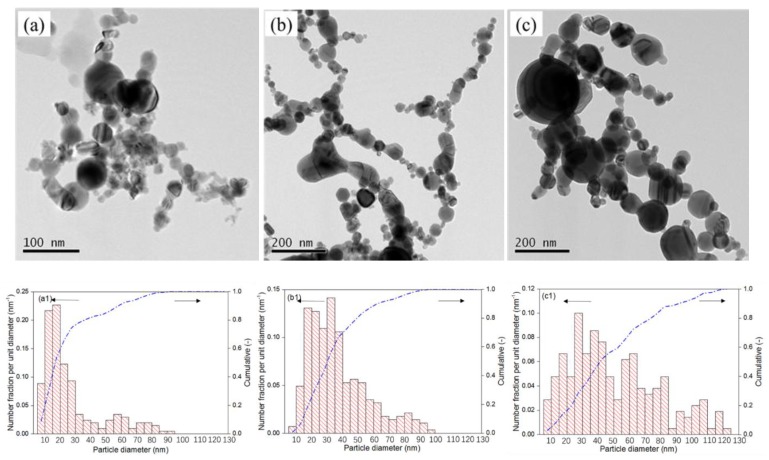
TEM images of nickel nanoparticle in argon arc in different concentrations of hydrogen. (**a**–**c**) correspond to hydrogen concentrations of 0, 30, and 50 vol%, respectively. (**a1**–**c1**) represent the particle size distributions.

Figure 7 indicates the TEM images of nickel nanoparticles obtained at different hydrogen concentrations from 0 vol% to 50 vol%. Figure 7a shows that nanoparticles are dominated by a spherical shape besides some irregular shapes when the hydrogen concentration is 0 vol%. Figure 7b,c illustrate that all the nanoparticles are spherical. In comparison with nanoparticles obtained in diffuse and multiple modes, the particle size becomes larger in constricted mode. Figure 7a1–c1 reveal the corresponding particle size distributions. The mean diameters of nanoparticles are 20, 33, and 63 nm, corresponding to the hydrogen concentrations of 0, 30, and 50 vol%, respectively. As the evaporation rate of nickel anode increases, the average particle size increases with the increase of hydrogen concentration. The particle diameter is strongly affected by the number species of nickel vapor. A high nickel vapor concentration would enhance the nanoparticle growth by condensation, resulting in large particle size distribution. In the case of higher hydrogen concentration, the evaporation of nickel metal was enhanced by thermal pinch effect. According to the formation mechanism of nanoparticles by arc discharge (Figure S2), the metal vapor will grow into nanoparticles by condensation and collision after nucleation. Higher concentration of metal vapor leads to larger particle size by condensation. Therefore, the higher hydrogen concentration contributes to larger average particle size. The nanoparticle size can be controlled by the following strategies, modification of the power supply by using capacitor or coil, using shield gas around the cathode [10], selecting plasma gas which contributes to form constricted arc, using hollow cathode [12] and rotating anode [7].

**Figure 8:**
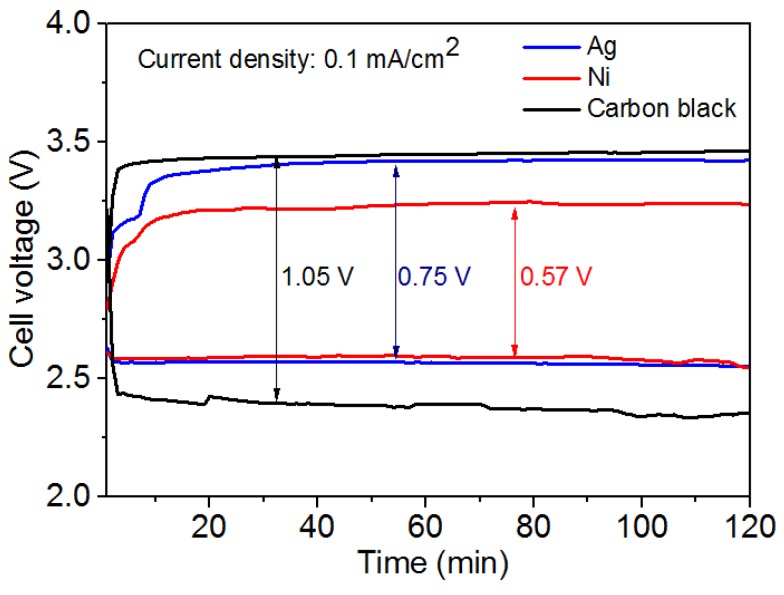
Charge-discharge profiles of nickel nanoparticles as catalysts for hybrid Na-air battery compared with commercial silver nanoparticles and carbon black at the current density of 0.1 mA/cm^2^.

Figure 8 presents the discharge-charge curves of the hybrid Na-air battery with nickel nanoparticles, silver nanoparticles, and carbon black catalysts. The current density is 0.1 mA/cm^2^ and the discharging-charging time is 2 h. Figure 8 shows that the battery using carbon black as catalyst displayed a discharge voltage of 2.38 V, corresponding to 76.53% of the theoretical voltage. Carbon black has a relatively poor catalytic performance due to its limited specific surface area and few activation sites [23]. The hybrid Na-air battery using silver nanoparticles has a discharge voltage of 2.63 V. Notably, a high charge voltage of 3.38 V is obtained due to the limitation of the catalytic performance of OER, resulting in a voltage gap of 0.75 V. Nevertheless, the hybrid Na-air battery using nickel nanoparticles as catalysts displays a higher discharge voltage of 2.65 V and lower charge voltage of 3.22 V than that using commercial silver nanoparticle, which results in a lower voltage gap of 0.57 V, corresponding to a round-trip efficiency of 80.3%. As shown in Figure S3, nickel nanoparticles show better electrochemical performances at different discharge current densities than Ag nanoparticles and carbon black. The good catalytic performances of nickel nanoparticles prepared by DC arc in 50% hydrogen concentration are ascribed to good conductivity and additional activation sites induced by good crystallinity and appropriate particle size distribution. Moreover, nickel nanoparticles may be polycrystalline, and the surfaces seen from TEM images are covered with amorphous NiO layers (Figure S4a). The NiO layer was also confirmed by XPS results in Figure S5. NiO has typical peaks for Ni^2+^ in NiO: 855.3 eV for Ni^2+^ 2p3/2 [24,25]. The composite of Ni/NiO contributes to their good catalytic performances for OER and ORR by the low charge transfer resistance of NiO [26].

**Figure 9:**
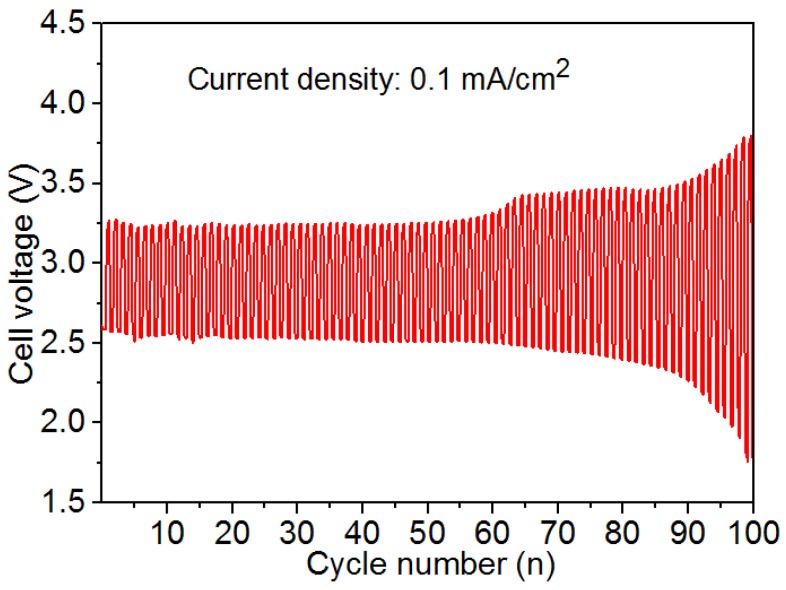
Cycling performance of the hybrid Na-air battery with nickel nanoparticles as catalysts during 100 cycles at a current density of 0.1 mA/cm^2^.

Figure 9 illustrates the cycling performance of the hybrid Na-air battery with nickel nanoparticles as catalysts. The battery cycles for 20 min at the current density of 0.1 mA/cm^2^ of discharging and charging per cycle. The high charge-discharge efficiency and good durability for Ni nanoparticles as catalysts during 100 cycles are attributed to two reasons. First, Ni nanoparticles with good conductivity, additional activation sites, and Ni/NiO composite structure contribute to their good catalytic performances. Second, the appropriate particle size distribution leads to the good stability of nickel nanoparticles as catalysts in the hybrid Na-air battery. During the first 50 charge-discharge processes, the tripping efficiency decreases from 80.3% to 80.0%. This result indicates that the hybrid Na-air battery using nickel nanoparticles as catalysts has a good cycling performance [21]. Nevertheless, from the 50th to the 100th charge-discharge process, the round-trip efficiency drops from 80.0% to 69.3% due to the evaporation of NaOH. The limited volume of NaOH solution is one restricted factor in this experiment (around 0.2 mL of NaOH) and leads to an increase in the NaOH concentration, even with the separation of the electrolyte and catalyst between the aqueous solutions [19]. The high solubility of oxygen under alkaline condition and the increase of the NaOH concentration enhance the internal resistance of the battery [20]. 

## 4. Conclusions

Nickel nanoparticles were prepared by the DC arc discharge method. Argon and argon/hydrogen mixtures were used as plasma gas. The hydrogen concentrations of 0, 30, and 50 vol% in the argon arc correspond to diffuse, multiple, and constricted arc-anode attachments, respectively. We investigated the effects of hydrogen concentration on the formation of different arc-anode attachment modes, understanding the arc phenomena contributes to the effective preparation of nanoparticles. Nickel nanoparticle generated by DC arc discharge in 50 vol% H_2_ concentration has high productivity, fine crystallinity, and appropriate size distribution. The large evaporation of the anode material leads to the formation of constricted anode attachment in the case of high hydrogen concentration. The variation waveform of the cathode jet follows the arc current shape in multiple arc-anode attachment modes at 30 vol% of hydrogen. Meanwhile, no clear relationship is found between the arc current and the area of the anode jet in the constricted mode. In comparison with nanoparticles obtained in diffuse and multiple modes, the particle size becomes larger in the constricted mode due to the large metal vapor concentration. The mean diameters of nanoparticles are 20, 33, and 63 nm, corresponding to the diffuse, multiple, and constricted arc-anode attachments, respectively. 

Nickel nanoparticles generated by 50 vol% of hydrogen concentration were used as catalysts for a hybrid sodium–air battery. Nickel nanoparticles, owing to their good crystallinity, additional activation sites, and Ni/NiO composite structured, have relatively better catalytic performance than commercial silver nanoparticles and carbon black. Nickel nanoparticles used as catalysts display 0.57 V voltage gap at the current density of 0.1 mA/cm^2^, which is lower than silver nanoparticles and carbon black corresponding to 0.75 V and 1.05 V, respectively. Nickel nanoparticles demonstrate good stability like the catalysts in hybrid sodium-air battery. As nickel nanoparticles possess a lower cost than silver nanoparticles and other noble metals, nanoparticles generated by arc discharge method are promising catalysts for hybrid sodium-air batteries and other alkaline metal-air batteries used in large-scale applications. 

## Supplementary Materials

The following are available online at http://www.mdpi.com/2079-4991/8/9/684/s1, Figure S1: (a) Schematic of a typical hybrid Na-air battery, (b) structure of the catalyst layer; Figure S2: The formation mechanism of metal nanoparticle by arc discharge method; Figure S3: (a) Discharge voltage profiles of hybrid Na-air batteries with Ni nanoparticles, Ag nanoparticles and carbon black as catalyst at different current densities. (b) Current density dependence of discharge voltage for batteries with different catalysts. The plots correspond to the measured data, and the solid lines indicate the fitted spectra; Figure S4: (a) TEM image of the Ni nanoparticles. (b) SAED pattern for the Ni nanoparticles; Figure S5: XPS spectra of Ni nanoparticles presented by arc discharge method.
