# Supplementary material for: Preparation of Nickel Nanoparticles by Direct Current Arc Discharge Method and Their Catalytic Application in Hybrid Na-Air Battery
Source: Nanomaterials (Basel). 2018 Sep 1;8(9):684. doi: 10.3390/nano8090684 (PMC6165376; doi:10.3390/nano8090684)
Supplement: Supplementary file 1 [file nanomaterials-08-00684-s001.pdf]

Supporting information

Preparation of Nickel Nanoparticles by Arc Discharge Method and Their Catalytic Application in Hybrid Na-Air Battery

Fengmei Su[1], Xuechao Qiu[1], Feng Liang[1,2*], Manabu Tanaka[3], Tao Qu[1,2], Yaochun Yao[1], Wenhui Ma[1,2], Bin Yang[1,2], Yongnian Dai[1,2], Katsuro Hayashi[4], Takayuki Watanabe[3] 1 Faculty of Metallurgical and Energy Engineering, Kunming University of Science and Technology, Kunming 650093, China

2 State Key Laboratory of Complex Nonferrous Metal Resources Clear Utilization, Kunming University of Science and Technology, Kunming 650093, China

3 Department of Chemical Engineering, Kyushu University, Fukuoka 819-0395, Japan

4 Department of Applied Chemistry, Faculty of Engineering, Kyushu University, Fukuoka 819-0395, Japan

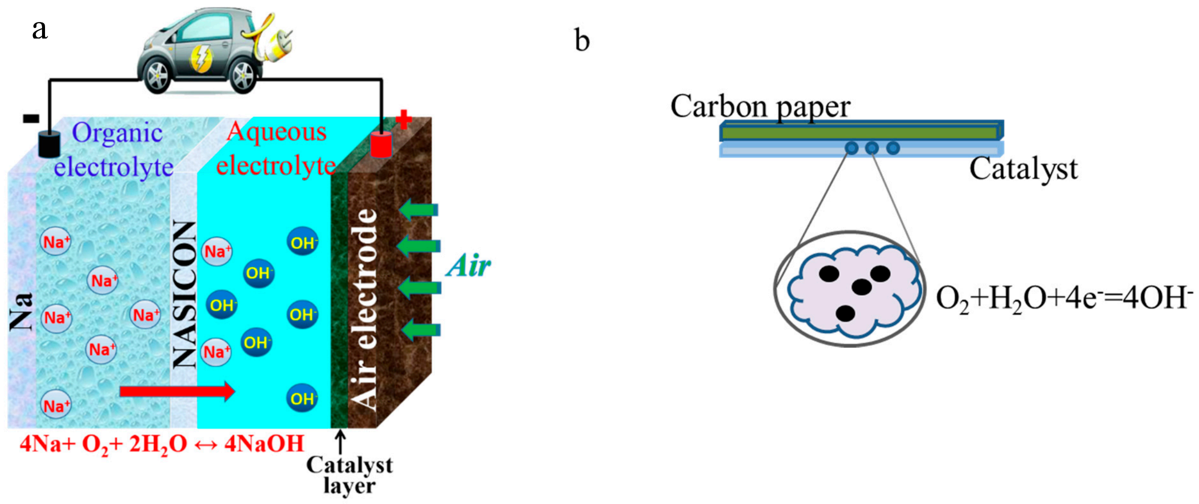

Fig.s1(a) Schematic of a typical hybrid Na-air battery, (b) structure of the catalyst layer .

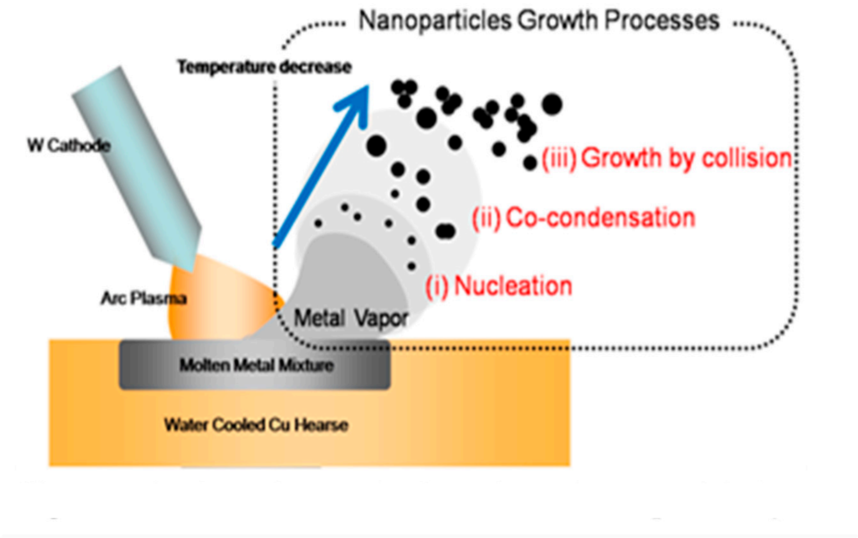

Fig. s2 The formation mechanism of metal nanoparticle by arc discharge method.

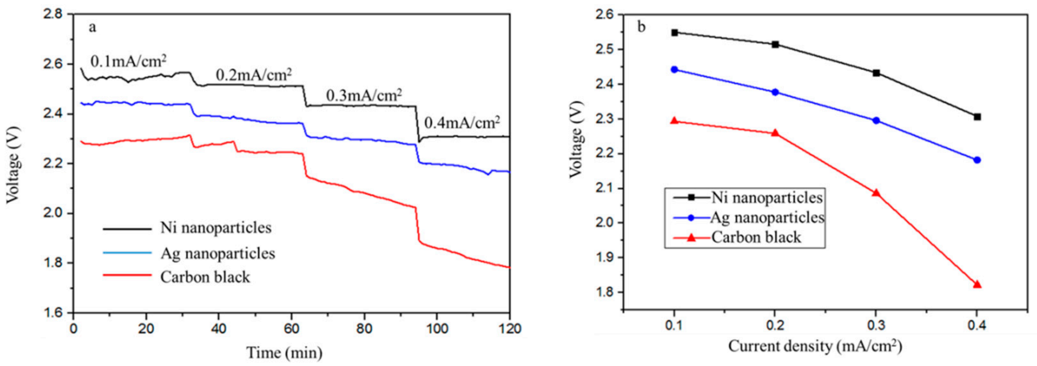

Fig. s3 (a) Discharge voltage profiles of hybrid Na-air batteries with Ni nanoparticles, Ag nanoparticles and carbon black as catalyst at different current densities. (b) Current density dependence of discharge voltage for batteries with different catalysts. The plots correspond to the measured data, and the solid lines indicate the fitted spectra.

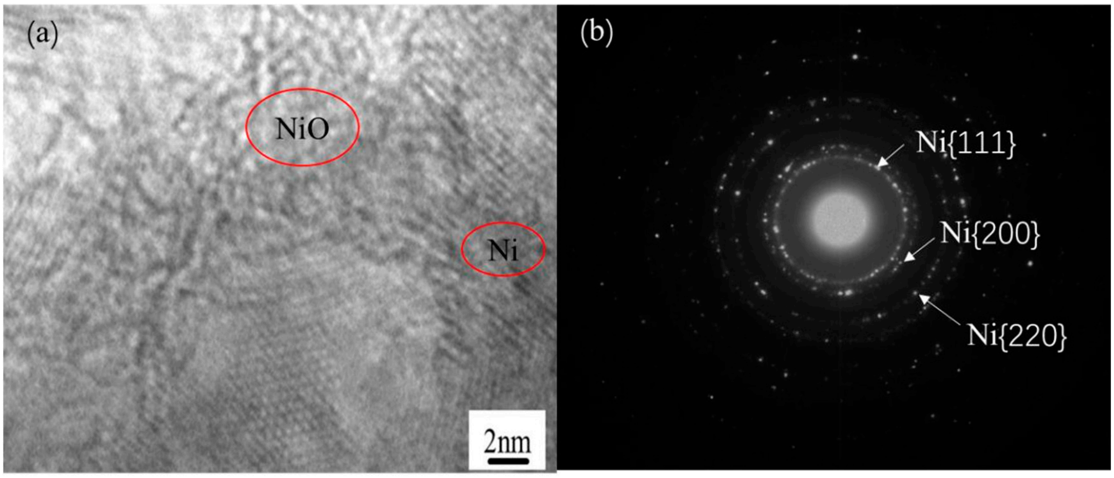

Fig. s4. (a) TEM image of the Ni nanoparticles. (b) SAED pattern for the Ni nanoparticles.

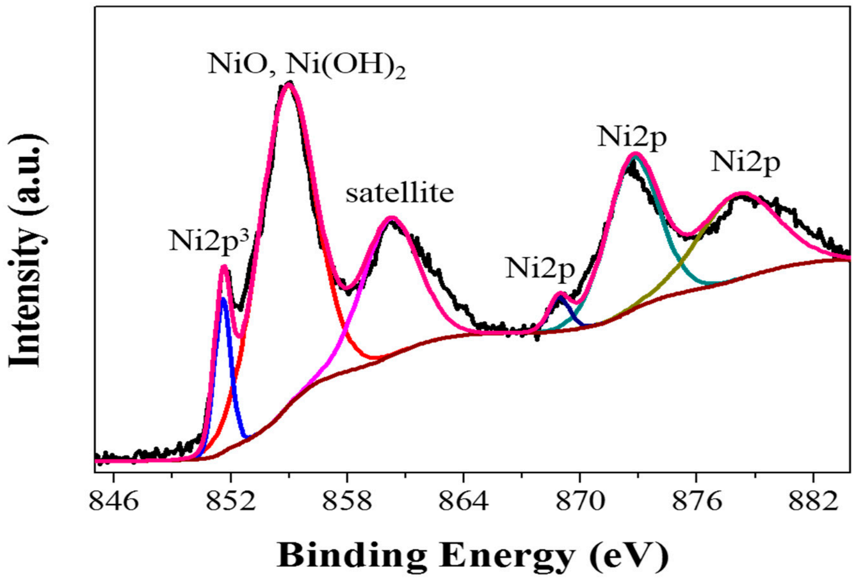

Fig.s5 XPS spectra of Ni nanoparticles presented by arc discharge method.
